# EMB3/389: Information Systems for Evidence-based Healthcare: status, issues and perspectives

**DOI:** 10.2196/jmir.1.suppl1.e23

**Published:** 1999-09-19

**Authors:** N Jayaram

**Affiliations:** ^1^University of Westminster, HarrowUK

**Keywords:** Evidence-based Healthcare, Healthcare Information Systems, Maturity Level

## Abstract

**Introduction:**

In the United Kingdom, the National Health Service (NHS) has embarked on the formation of health action zones for the delivery of good quality community-centric healthcare. It is recognised that the evidence-based healthcare practice is critical in this venture, and the optimistic projection is that community-centric healthcare encompassing medical, dental, nursing, health visiting, optical and pharmaceutical services will be brought under the evidence-based healthcare practice framework. The evidence-based medicine and its practice in its 'first wave' with its six steps provided a paradigm shift in the clinical practice by integrating experiential and external evidence with clinical practice to arrive at clinical decisions and establish disease management framework. The internet was rightly considered as the key player in the evidence-based healthcare practice. The shortcomings of this global system has constrained the wide-spread application of the evidence-based practice.

**Methods:**

We will first take an in-depth look at the relevance, quality and accessibility issues, by presenting examples from respiratory and metabolic disease domains, particularly from the classes of paediatric metabolic disorders. While analysing the information presented, we will also assess to what degree the information needs of clinicians as well as patient/home-carers are met. We will examine what is required in terms of structure, protocols, information storage and access to meet the two often disparate needs.

**Results:**

We will argue that the information should be in a form that enables their effective assimilation thereby enhancing the usage of the evidence-based practice. Concepts of componentware, model-based information- bayesian causal graphs will presented with examples. Examples of emerging database implementations will be referred to in these examples. Our on-going research work on MEDINFONET, and models of domain-specific software agents for knowledge management will be presented.

**Discussion:**

The service-centric model of the next generation internet presents a bright perspective. From the evidence-based healthcare practice point-of-view, its differential service (diff-serv) facility with emphasis on classes of services will largely meet the speed of access requirement. The 'object web' with data integration facility, digital libraries, domain-specific search agents etc.. will provide a better information quality. We argue that models and metrics for information systems and its environment are needed, and in this connection present a five-layer maturity model for evidence-based healthcare practice.

